# ICAR ATRP of Acrylonitrile under Ambient and High Pressure

**DOI:** 10.3390/polym8030059

**Published:** 2016-03-02

**Authors:** Zhicheng Huang, Jing Chen, Lifen Zhang, Zhenping Cheng, Xiulin Zhu

**Affiliations:** Suzhou Key Laboratory of Macromolecular Design and Precision Synthesis, Jiangsu Key Laboratory of Advanced Functional Polymer Design and Application, Department of Polymer Science and Engineering, College of Chemistry, Chemical Engineering and Materials Science, Soochow University, Suzhou 215123, China; huangzc_1991@163.com (Z.H.); chenjing_sep@163.com (J.C.); xlzhu@suda.edu.cn (X.Z.)

**Keywords:** ICAR ATRP, ambient pressure, high pressure, PAN

## Abstract

It is well known that well-defined polyacrylonitrile (PAN) with high molecular weight (*M*_w_ > 106 g·mol^−1^) is an excellent precursor for high performance carbon fiber. In this work, a strategy for initiators for a continuous activator regeneration atom transfer radical polymerization (ICAR ATRP) system for acrylonitrile (AN) was firstly established by using CuCl_2_·2H_2_O as the catalyst and 2,2′-azobis(2-methylpropionitrile) (AIBN) as the thermal initiator in the presence of ppm level catalyst under ambient and high pressure (5 kbar). The effect of catalyst concentration and polymerization temperature on the polymerization behaviors was investigated. It is important that PAN with ultrahigh viscosity and average molecular weight (*M*_η_ = 1,034,500 g·mol^−1^) could be synthesized within 2 h under high pressure.

## 1. Introduction

**Scheme 1:**
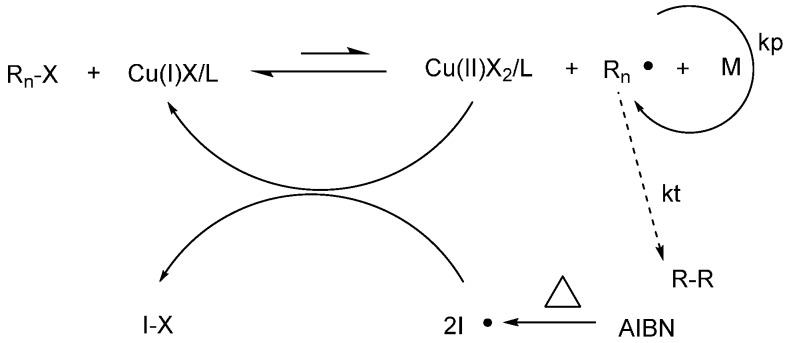
The proposed mechanism of initiators for continuous activator regeneration atom transfer radical polymerization (ICAR ATRP).

In the past decades, we have witnessed great progress of ”living”/controlled radical polymerization (LRP) in terms of the ability of macromolecular design and precision synthesis. Especially atom transfer radical polymerization (ATRP) [1,2,3,4], as one of the most efficient and robust reversible deactivation radical polymerization (RDRP) methods, has developed various ATRP techniques to improve synthetic methods of polymers. These techniques include reverse ATRP [5,6,7,8,9], initiators for continuous activator regeneration atom transfer radical polymerization (ICAR ATRP) [10,11,12,13,14,15,16,17,18] which apply traditional radical initiators and the higher oxidation state of the transition metal complex (the proposed mechanism shown in Scheme 1), activators generated by electron transfer ATRP (AGET ATRP) [19,20,21] which use reducing agents to replacing traditional radical initiators, e-ATRP [22,23,24,25] which employs the electrochemical method, photoinduced organic catalyzed metal-free ATRP [26,27,28,29], and the very recently developed metal-free photoinduced electron transfer–atom transfer radical polymerization (PET–ATRP) [30].

It is noteworthy that the radical chain propagation reaction was strengthened with the distinctly increased polymerization rate coefficient, while the chain termination reaction was suppressed under high pressure inherently due to diffusional limitations [31,32,33,34]. Therefore, the high pressure technique [35,36,37] is a charismatic means in ultrahigh molecular weight polymer synthesis. Penelle’s group [38] prepared high molecular weight (*M*_w_ > 1000 kg·mol^−1^) and narrow molecular weight distribution (*M*_w_/*M*¬_n_ = 1.03) polymethyl methylacrylate (PMMA) under 5 kbar. Fukuda and his partner [39] have depicted that PMMA (*M*_w_ > 3600 kg·mol^−1^, *M*_w_/*M*¬_n_ = 1.24) was obtained by conventional ATRP. Furthermore, ultrahigh molecular weight (*M*_w_ > 1000 kg·mol^−1^) and low molecular distribution (*M*_w_/*M*¬_n_ = 1.25) polystyrene (PS) have been processed using AGET ATRP at ambient temperature under 6 kbar in Matyjaszewski’s group [40].

It is well known that well-defined polyacrylonitrile (PAN) [41,42,43,44] with stereo-tacticity and high molecular weight (*M*_w_ > 106 g·mol^−1^) is an excellent precursor for high performance carbon fiber [45,46,47] and is an effective component of mesoporous carbon material [48] in photoelectrical device applications. Unfortunately, most of the commercially available PAN with high molecular weight (*M*_w_ < 1000 kg·mol^−1^) and high molecular weight distribution (*M*_w_/*M*¬_n_ > 3.0) was obtained by conventional free radical polymerization, which was unsatisfactory for employing as a precursor for high quality carbon-based materials. Therefore, could we obtain ultrahigh molecular weight and narrow molecular weight distribution PAN with the aid of the RDRP method under high pressure?

**Scheme 2:**
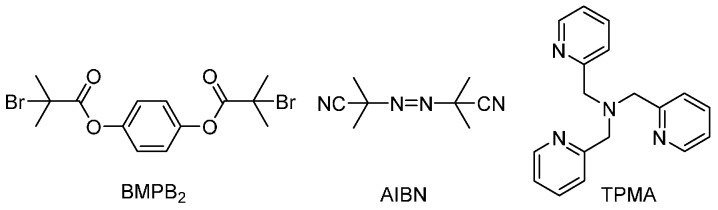
The structure of chemicals used in this work.

In this work, inspired by the enhancement of the polymerization rate and the suppression of chain termination under high pressure, we tried to synthesize well-defined PAN with high molecular weight and narrow molecular distribution. Herein, an ICAR ATRP system was employed to conduct the polymerization of AN under ambient and high pressure (5 kbar) by using a highly efficient catalyst system, CuCl_2_·2H_2_O/tris(2-pryidylmethyl)amine (TPMA) [49]. At the same time, a difunctional initiator, 1,4-phenylene bis(2-bromo-2-methypropanoate) (BMPB_2_) (Scheme 2 for the structure) [50], was used in this work with 2,2′-azobis(2-methylpropionitrile) (AIBN) as the traditional thermolysis radical initiator. The effects of catalyst concentration and polymerization temperature were investigated.

## 2. Experimental Section

### 2.1. Materials

Acrylonitrile (AN, +99%, Shanghai Chemical Reagents Co. Ltd., Shanghai, China) was purified by passing through a neutral alumina column before use. *N,N*-Dimethylformamide (DMF, analytical reagent, Shanghai Chemical Reagents Co. Ltd.) and dimethylsulfoxide (DMSO, analytical reagent, Shanghai Chemical Reagents Co. Ltd.) were dried using 4 Å molecular sieve. The 2,2′-azobis(2-methylpropionitrile) (AIBN, chemical pure, Shanghai Chemical Reagents Co. Ltd.) were recrystallized from ethanol, and dried in a vacuum oven. Tris(2-pryidylmethyl)amine (TPMA, +98%) was purchased from Shanghai Xiarui Trade Co. Ltd. (Shanghai, China), and CuCl_2_·2H_2_O (98%, Sigma-Aldrich, Shanghai, China) were purchased from Shanghai Chemical Reagents Co. Ltd. and used as received. The initiator 1,4-phenylene bis(2-bromo-2-methypropanoate) (BMPB_2_) was synthesized according to the literature [51]. All the other chemicals were obtained from Shanghai Chemical Reagents Co. Ltd. and used as-received unless mentioned otherwise.

### 2.2. Typical Procedure for Initiators for a Continuous Activator Regeneration Atom Transfer Radical Polymerization (ICAR ATRP) of Acrylonitrile (AN) under Ambient or High Pressure

A typical ICAR ATRP procedure with the molar ratio of [AN]_0_:[BMPB_2_]_0_:[CuCl_2_·2H_2_O]_0_:[TPMA]_0_:[AIBN]_0_ = 500:1:0.05:0.1:0.2 was carried out in a clean ampoule (10 mL) (under atmospheric pressure) or a bag made by polyperfluorinated ethylene propylene film (under high pressure). The reaction mixture was prepared by adding AN (1.50 mL, 22.80 mmol), BMPB_2_ (18.60 g, 4.56 × 10^−2^ mmol), CuCl_2_·2H_2_O (0.38 mg, 2.81 × 10^−3^ mmol), TPMA (1.32 mg, 4.56 × 10^−3^ mmol), AIBN (1.50 mg, 9.13 × 10^−3^ mmol) and DMSO (3.0 mL). The solution was bubbled with argon for about 20 min to eliminate the dissolved oxygen. Then, the ampoule was flame-sealed and placed in an oil bath at 60 °C. The bag was sealed by plastic-envelop machine and placed in a water bath with high pressure (5 kbar). The high pressure reaction system (HPP. L2-600/0.6) was purchased from Tianjin Huatai Sen Miao Biological Engineering Technology Co. Ltd. (Tianjin, China). The reactor includes a hydraulic piston-cylinder unit with pressure reaction vessel equipped with a temperature controller and a pressure sensor using water as the medium of pressure conductivity. The ampoule or a bag was opened after the desired polymerization time under atmospheric pressure and high pressure, respectively. The mixture was dissolved in a certain amount of DMF and precipitated into 250 mL of methanol. The polymers were isolated by suction filtering in a Buchner funnel and dried in vacuum oven until a constant weight at 35 °C. The monomer conversion was obtained gravimetrically.

### 2.3. Characterizations

The ^1^H NMR spectra of the obtained polymers were recorded on a Bruker 300 MHz nuclear magnetic resonance instrument using CDCl_3_ as the solvent and tetramethylsilane (TMS) as an internal standard. The number-average molecular weight (*M*_n,GPC_) and molecular weight distribution (*M*_w_/*M*_n_) values of the resultant polymers determined using a TOSOH HLC-8320 gel permeation chromatograph (GPC) equipped with a refractive-index detector (Tosoh Bioscience Shanghai Co. Ltd., Shanghai, China), using TSKgel guard column SuperMP-N (4.6 mm × 20 mm) and two TSK gel SupermultiporeHZ-N (4.6 mm × 150 mm) with measurable molecular weight ranging from 10^2^ to 10^6^ g/mol. DMF with 0.5 mmol/L of LiBr was used as eluent at a flow rate of 1.0 mL/min at 40 °C. GPC samples were injected using a TOSOH plus autosampler and calibrated with PMMA standards purchased from TOSOH.

## 3. Results and Discussion

### 3.1. Effect of Catalyst Concentration on ICAR ATRP of AN under Ambient Pressure

The catalyst concentration of Cu(II)-catalyzed ICAR ATRP was firstly investigated under ambient pressure, and the results are shown in Table 1. With the increasing of Cu(II) catalyst concentration, a narrower molecular weight distribution was observed (*M*_w_/*M*_n_ = 1.20, 250 ppm catalyst, Entry 1 in Table 1), while *M*_w_/*M*_n_ = 1.80 (2.5 ppm catalyst, Entry 4 in Table 1), which indicated that a too-low catalyst concentration leads to a unsuccessful controlled polymerization. Because PAN standards are unavailable, the number-average molecular weight values determined by GPC with PMMA standards are much larger than the exact molecular weight values. Therefore, the calibrated number-average molecular weights (*M*_n,GPC_/2.5 as proposed by Matyjaszewski [52]) were adopted, which were consistent with the corresponding theoretical ones.

### 3.2. Effect of Temperature on ICAR ATRP of AN under Ambient and High Pressure

It is well known that temperature plays a vital role in controlled polymerization systems. As shown in Table 2, well-defined PANs were obtained in all cases, and the polymerization rate increased with temperature (from 30 to 60 °C) as expected. For example, 40.8% of monomer conversion was achieved within 120 h at 30 °C (Entry 4 in Table 2) while 61.9% of monomer conversion was obtained within 21 h at 60 °C (Entry 1 in Table 2). Similar results (Table 3) were also observed for the polymerizations under high pressure (5 kbar); however, the polymerization rate was enhanced significantly under high pressure. For instance, 51.3% of monomer conversion was obtained within 7 h at 40 °C under 5 kbar (Entry 3 in Table 3) while 45 h was needed to reach 43.5% of monomer conversion at 40 °C under ambient pressure (Entry 3 in Table 2).

### 3.3. Polymerization Kinetics and Chain-End Analysis

**Figure 1:**
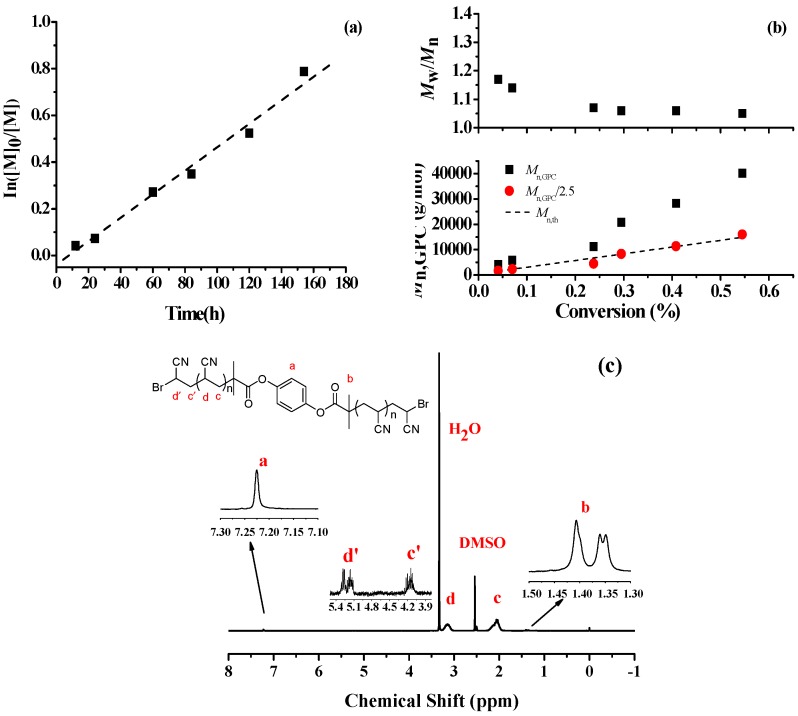
ln([M]_0_/[M]) as a function of time (**a**) and number-average molecular weight and molecular weight distribution (*M*_w_/*M*_n_) *versus* monomer conversion (**b**) for the initiators for a continuous activator regeneration atom transfer radical polymerization (ICAR ATRP) of acrylonitrile (AN) under ambient pressure; (**c**) The ^1^H NMR spectrum of polyacrylonitrile (PAN) (*M*_n,GPC_ = 28,400 g·mol^−1^, *M*_w_/*M*_n_ = 1.06, conversion = 40.8%) with dimethylsulfoxide (DMSO) as the solvent and tetramethylsilane (TMS) as the internal standard. Polymerization conditions: [AN]_0_:[BMPB_2_]_0_:[CuCl_2_·2H_2_O]_0_:[TPMA]_0_:[AIBN]_0_ = 500:1:0.01:0.1:0.2, *V*_AN_ = 1.5 mL, *V*_DMSO_ = 3.0 mL, *T* = 30 °C under ambient pressure.

In order to verify the “living” feature of this polymerization system, polymerization kinetics of Cu(II)-catalyzed ICAR ATRP of AN under ambient pressure were investigated as depicted in Figure 1. The pseudo-first-order polymerization kinetic plots (Figure 1a), which indicated a constant radical concentration through the polymerization period, and approximately linear evolution of the calibrated number-average molecular weight (*M*_n,GPC_/2.5) close to the corresponding theoretical values and narrower molecular weight distribution (*M*_w_/*M*_n_ < 1.20) with the monomer conversion (Figure 1b) were observed. In addition, the chain-end of PAN (*M*_n,GPC_ = 28,400 g·mol^−1^, *M*_w_/*M*_n_ = 1.06) prepared by ICAR ATRP under ambient pressure was analyzed by ^1^H NMR spectroscopy as shown in Figure 1c. The peaks of a (δ = 7.23 ppm) and b (δ = 1.41 and 1.35 ppm) were attributed to the protons of phenyl and methyl of BMPB_2_, respectively. Moreover, the peaks of d (δ = 3.20 ppm) and c (δ = 2.11 ppm) corresponded to the methylene protons and methyl protons in the PAN backbone. Because of the electron-attracting function of the Br atom, the signals of peak d′ (δ = 5.21~5.32 ppm) and c′ (δ = 4.15 ppm) were attributed to the methylene protons and methyl protons of the last repeat unit in the PAN chains. All of the above results confirmed the “living” feature of this ICAR ATRP system.

### 3.4. Synthesis of High Molecular Weight Polyacrylonitrile (PAN)

In order to obtain high molecular weight PAN, a large molar ratio of AN to BMPB_2_ (up to 20,000:1) was used to conduct Cu(II)-catalyzed ICAR ATRP under ambient and high pressure, respectively, and the results are shown in Table 4. Under ambient pressure (Entries 1 and 2 in Table 4), 25.3% of monomer conversion was achieved (Entry 2 in Table 4) within 9 h (further increasing the polymerization time, the monomer conversion almost kept constant), and the corresponding molecular weight of PAN was up to 260,700 g·mol^−1^ (*M*_w_*/M*_n_ = 1.20). By comparison, under high pressure (5 kbar), the monomer conversion could reach up to 72.3% within 2 h (Entry 4 in Table 4). However, due to the corresponding ultrahigh molecular weight PAN, the number-average molecular weight and molecular weight distribution values were not available by GPC, and therefore the viscosity-average molecular weight values (*M*_η_) were determined by the Mark–Houwink equation [53]. A high molecular weight up to 1,034,500 g·mol^-1^ was obtained with the large molar ratio of [AN]_0_:[BMPB_2_]_0_ = 20,000:1. Obviously, the high pressure polymerization technique would be more suitable for ultrahigh molecular weight polymer synthesis as compared to polymerization under atmosphere.

## 4. Conclusions

A “living”/controlled radical polymerization system for ICAR ARTP of AN using CuCl_2_·2H_2_O as the catalyst, TPMA as the efficient ligand, and BMPB_2_ as the difunctional initiator was successfully established in the presence of ppm level catalyst. In addition, PAN with an ultrahigh viscosity-average molecular weight up to *M*_η_ = 1,034,500 g·mol^−1^ could be synthesized just within 2 h under high pressure by Cu(II)-catalyzed ICAR ATRP of AN. It is particularly noteworthy that an enormous advantage in terms of the polymerization rate under high pressure in comparison with that under atmosphere was displayed. Therefore, the high pressure polymerization technique applied to ultrahigh molecular weight polymer synthesis is promising.

## Figures and Tables

**Table 1:** Effect of initial Cu(II) concentration on the polymerization of acrylonitrile (AN) at ambient pressure ^a^.

Entry	x	Cu (ppm)	Time (h)	Conv. (%)	*M*_n,th_^b^ (g/mol)	*M*_n,GPC_/2.5 (g/mol)	*M*_w_/*M*_n_
1	0.5	250	23	44.9	47,600	47,600	1.20
2	0.1	50	9	36.5	38,600	50,400	1.28
3	0.02	10	9	74.9	79,400	69,640	1.33
4	0.005	2.5	9	37.7	40,000	85,560	1.80

^a^ Polymerization conditions: [AN]_0_:[BMPB_2_]_0_:[CuCl_2_·2H_2_O]_0_:[TPMA]_0_:[AIBN]_0_ = 2000:1:x:0.3:0.3 (x = 0.5, 0.1, 0.02, and 0.005), *V*_AN_ = 1.5 mL, *V*_DMSO_ = 3.0 mL, T = 60 °C; ^b^ *M*_n,th_ = ([M]_0_/[BMPB_2_]_0_) × *M*_w,AN_ × Conv.%.

**Table 2:** Effect of polymerization temperature on initiators for a continuous activator regeneration atom transfer radical polymerization (ICAR ATRP) of AN at ambient pressure ^a^.

Entry	Time (h)	T (°C)	Conv. (%)	*M*_n,th_^b^ (g/mol)	*M*_n,GPC_/2.5 (g/mol)	*M*_w_/*M*_n_
1	21	60	61.9	16,400	18,200	1.06
2	22	50	54.7	14,500	16,880	1.10
3	45	40	43.5	11,500	14,040	1.06
4	120	30	40.8	10,800	11,300	1.06

^a^ Polymerization conditions: [AN]_0_:[BMPB_2_]_0_:[CuCl_2_·2H_2_O]_0_:[TPMA]_0_:[AIBN]_0_ = 500:1:0.01:0.1:0.2, V_AN_ = 1.5 mL, V_DMSO_ = 3.0 mL; ^b^ *M*_n,th_ = ([M]_0_/[BMPB_2_]_0_) × *M*_w,AN_ × Conv.%.

**Table 3:** Effect of polymerization temperature on ICAR ATRP of AN under high pressure ^a^.

Entry	Time (h)	T (°C)	Conv. (%)	*M*_n,th_^b^ (g/mol)	*M*_n,GPC_/2.5 (g/mol)	*M*_w_/*M*_n_
1	1	60	45.1	11,900	13,880	1.14
2	4	50	41.7	11,000	13,120	1.13
3	7	40	51.3	13,600	16,640	1.11
4	8	30	21.3	5,600	7,520	1.12

^a^ Polymerization conditions: [AN]_0_:[BMPB_2_]_0_:[CuCl_2_·2H_2_O]_0_:[TPMA]_0_:[AIBN]_0_ = 500:1:0.01:0.1:0.2, V_AN_ = 1.5 mL, V_DMSO_ = 3.0 mL; ^b^ *M*_n,th_ = ([M]_0_/[BMPB_2_]_0_) × *M*_w,AN_ × Conv.%.

**Table 4:** Synthesis of high molecular weight PAN under high and ambient pressure ^a^.

Entry	x	Conv. (%)	*M*_n,th_^d^ (g/mol)	*M*_η_^e^ (g/mol)	*M*_n,GPC_/2.5 (g/mol)	*M*_w_/*M*_n_
1 ^b^	10,000	32.1	169,900	–	190,200	1.18
2 ^b^	20,000	25.3	267,800	–	260,700	1.20
3 ^c^	10,000	81.4	431,600	541,200	–	–
4 ^c^	20,000	72.3	766,400	1,034,500	–	–

^a^ Polymerization conditions: [AN]_0_:[BMPB_2_]_0_:[CuCl_2_·2H_2_O]_0_:[TPMA]_0_:[AIBN]_0_ = x:1:0.1:0.3:0.3 (x = 1000 and 2000), V_AN_ = 1.5 mL, V_DMSO_ = 3.0 mL, T = 65 °C; ^b^ Under ambient pressure, polymerization time = 9 h; ^c^ Under 5 kbar, polymerization time = 2 h; ^d^ *M*_n,th_ = ([M]_0_/[BMPB_2_]_0_) × *M*_w,AN_ × Conv.%; ^e^ Mη calculated by the Mark–Houwink equation: [η] = 3.0 × 10^−2^ M_η_^0.75^.
